# Regulating the international surrogacy market:the ethics of commercial surrogacy in the Netherlands and India

**DOI:** 10.1007/s11019-020-09976-x

**Published:** 2020-09-14

**Authors:** Jaden Blazier, Rien Janssens

**Affiliations:** 1 Philosophy, Bioethics, and Health, Vrije Universiteit, Amsterdam, The Netherlands; 2 Dept. of Medical Humanities, Amsterdam University Medical Center, Location VU Medical Center, PO Box 7057, 1007MB Amsterdam, The Netherlands

**Keywords:** Ommercial surrogacy, Market, Exploitation, Commodification, Regulation

## Abstract

It is unclear what proper remuneration for surrogacy is, since countries disagree and both commercial and altruistic surrogacy have ethical drawbacks. In the presence of cross-border surrogacy, these ethical drawbacks are exacerbated. In this article, we explore what would be ethical remuneration for surrogacy, and suggest regulations for how to ensure this in the international context. A normative ethical analysis of commercial surrogacy is conducted. Various arguments against commercial surrogacy are explored, such as exploitation and commodification of surrogates, reproductive capacities, and the child. We argue that, although commodification and exploitation can occur, these problems are not specific to surrogacy but should be understood in the broader context of an unequal world. Moreover, at least some of these arguments are based on symbolic rhetoric or they lack knowledge of real-world experiences. In line with this critique we argue that commercial surrogacy can be justified, but how and under what circumstances depends on the context. Surrogates should be paid a sufficient amount and regulations should be in order. In this article, the Netherlands and India (where commercial surrogacy was legal until 2015) are case examples of contexts that differ in many respects. In both contexts, surrogacy can be seen as a legitimate form of work, which requires the same wage and safety standards as other forms of labor. Payments for surrogacy need to be high enough to avoid exploitation by underpayment, which can be established by the mechanisms of either minimum wage (in high income countries such as the Netherlands), or Fair-Trade guidelines (in lower-middle income countries such as India). An international treaty governing commercial surrogacy should be in place, and local professional bodies to protect the interests of surrogates should be required. Commercial surrogacy should be permitted across the globe, which would also reduce the need for intended parents to seek surrogacy services abroad.

## Introduction

Surrogacy occurs when a woman gestates and gives birth to a child for the intended parents.^1^ The surrogacy process is beneficial for parents who are unable to procreate, but it is riddled with ethical challenges. Where surrogacy is permitted, a subject of ongoing debate is the question of proper remuneration for surrogates. Surrogacy remuneration generally comes in two forms: altruistic (unpaid) surrogacy and commercial (paid) surrogacy. In an altruistic arrangement, the surrogate may not be compensated above and beyond expenses related to the pregnancy. The surrogate is entirely motivated by altruism, to help an infertile couple fulfill their wish for a child (Caelers 2001; Leeton et al. 1988). In a commercial arrangement, the surrogate is paid a fee on top of direct expenses. Often an agency is involved in matching up surrogates to intended parents and making sure the whole process runs smoothly. While most countries only allow altruistic surrogacy, or ban surrogacy altogether, commercial surrogacy is ever-present. Further, legislative and cost disparity between nations creates room for cross-border surrogacy (CBS), which is when people travel to other nations to access surrogacy services. While the debate about remuneration for surrogacy has been going on for a few decades, many countries have still not addressed the issue or are reconsidering their laws. Further, CBS has increased by large amounts in recent years (Merchant 2018), but international legislation (agreements, treaties, etc.) have not followed (Humbyrd 2009).

Both commercial and altruistic surrogacy have ethical drawbacks. Lack of payment could be exploitative (Wertheimer 1992; Wilkinson 2003) and could restrict reproductive autonomy (Andrews 1988; Lawrence 1991). However, paying someone to carry a child and subsequently give it up might also be wrong because of the possibility of exploitation by coercion (Wilkinson 2018) and commodification of reproductive labor, women, and children (Anderson 1990; Holder 1984; Radin 1987; Sandel 2013; Timms 2018). CBS exacerbates these ethical issues, especially when the intended parents are from a high-income country (HIC) and the surrogate is from a low- or middle-income country (LMIC). As predicted in Gena Corea’s 1985 dystopic novel The Mother Machine, CBS is criticized as a practice in which the bodies of poor women from developing countries are routinely instrumentalized for the benefit of richer people in the developed world, and for the profit of the global fertility industry (Gupta 2012). Most scholars agree that there are ethical issues with international commercial surrogacy, but whether or not the commercial aspect is the problem is up for debate (Spar 2005; Wilkinson 2003). Critics of commercial surrogacy tend to draw on negative imagery and symbolic rhetoric to paint a picture of commercial surrogacy as inherently unethical, without addressing ethical issues systematically (Andrews 1988). Meanwhile, ethical analyses of commercial surrogacy tend to make normative claims using theoretical, western ethics, without invoking analyses of the real-world experiences of surrogates in non-western countries (Bailey 2011). To find a middle ground between these extremes, we conduct an ethical analysis of this issue, taking into account the effect of context on what ethical remuneration would be. This provides insight into what kind of action is necessary from HICs, and LMICs, in order to mitigate ethical issues surrounding remuneration in the international surrogacy market. Following the publication of a letter of the Indian Ministry of Home Affairs in 2015, CBS was prohibited in India, but until then CBS was widely prevalent in the country. It is therefore, and also since there is more documentation on the Indian context than there is on other countries allowing CBS, that we choose India as case example, despite the 2015 ban.

### The Netherlands and India as case examples

The Netherlands is a classic example of a western country which prohibits commercial surrogacy, only allowing altruistic surrogacy under strict-conditions (Boele-Woelki and Vonk 2012). Despite the aversion of the government towards commercial surrogacy, there has been discussion about loosening restrictions in the Netherlands, in part to prevent Dutch intended parents from going abroad to access surrogacy. The 2016 report from the Staatscommissie Herijking Ouderschap (Government Committee on the Reassessment of Parenthood, from now on GCRP) included a proposal for legislative change, including a statutory framework for surrogacy, which would make surrogacy easier to do, and would allow surrogates to be paid a maximum of €500 per month (on top of expenses). In 2019, the Dutch minister of Legal Protection responded to the report, rejecting the suggestions from the GCRP for allowing paid surrogacy (“Dutch government reaction to recommendations of GCRP 2019; Ministry of Justice and Security. 2019). This demonstrates the ongoing uncertainty over whether or how much surrogates should be paid, in part because of the rise of CBS.

Up until 2015, India was a particularly popular destination for CBS. India legalized surrogacy in 2002 in order to promote surrogacy tourism, as a part of its growing market for medical tourism (Gupta 2012; Shetty 2012; Vincent and Aftandilian 2013). 13 years later, regulations were introduced and CBS was prohibited. Up until then, clinics were free to do as they wish (Shetty 2012). Earlier guidelines from the Ministry of Health and Family Welfare, which were put forth in 2008 by the Indian Council of Medical Research, were not binding and were accused of promoting ART rather than regulating it (Bailey 2011; Vincent and Aftandilian 2013). Up until recently, the profit-seeking mechanisms governing the fertility industry, in the context of widespread poverty, could have created a situation where exploitation and commodification of women were more likely (Vincent and Aftandilian 2013; Timms 2018). For these reasons, prohibitions on commercial surrogacy were suggested (Gupta 2012). In 2015 the ministry’s letter mentioned above effectively put an end to commercial surrogacy and CBS. In 2016 a ‘surrogacy regulation bill’ was issued by the Lok Sabha, the lower house of the Indian parliament. After the bill lapsed that same year, the Lok Sabha passed it in 2019. The bill now awaits passing by the Rajiya Sabha, the higher house of the parliament. Only after the higher house passes the bill will it become national law. It prohibits foreign nationals from commissioning surrogacy in India and exclusively reserves surrogacy for Indian, heterosexual, sub-fertile couples with a minimum of five years of marriage who will engage in an altruistic arrangement (Timms 2018). Below, we claim that it is not clear that the issues leading to the ban are inherently tied to the commercial aspect of surrogacy, demonstrating the need for a thorough ethical analysis. Instead of prohibiting commercial surrogacy and CBS outright, we propose regulations in order to prevent exploitation and commodification.

## Is commercial surrogacy inherently wrong? an ethical analysis

### The exploitation argument against commercial surrogacy

It is argued that paying women for surrogacy is exploitative. If it is exploitative, then, one uses a surrogate as a means unjustly or under conditions such that the surrogate does not consent (at least not validly) (Wertheimer 1992). For a surrogate to be unjustly used as a means, the effects on her welfare must be more negative than justice allows, which could mean the surrogate is harmed, or that she does not benefit sufficiently (Wilkinson 2003). Clearly harm to the surrogate is not the issue in this argument, since altruistic surrogacy is still seen as acceptable. Then, the surrogate may be unjustly used as a means if she is underpaid, which would occur if the physical and psychological risks to the surrogate are not properly compensated in relation to the benefit to the intended parents. This would mean the exploitation argument actually favors commercial surrogacy and higher payments to surrogates over altruistic surrogacy. But even with commercial surrogacy, exploitation by underpayment can happen. In India, the surrogate’s altruistic motivations and maternal duties used to be continually reiterated by surrogacy brokers to intimidate her to not demand higher payments or voice her concerns (Dabriak et al. 2007; Gupta 2012; Pande 2010). There was unequal bargaining power between the wealthier intended parents and the poor (and sometimes illiterate) surrogate (Lee 2009). Surrogacy contracts involved cross border clients and Indian slum-dwellers, giving rise to extreme polarization (Timms 2018). Regulations requiring sufficient, fair payments to surrogates would solve this problem.

Of course, the focus of the exploitation argument against commercial surrogacy is not that surrogates are underpaid. Exploitation, they argue, still occurs if the validity of consent is compromised by the coercive effect of the payment (Wilkinson 2003). If the pay is too high, there is a risk that surrogacy would become too attractive and poor women would become surrogates out of desperation for money (Brazier et al. 1998). This was particularly relevant in India where most Indian surrogates used to say that they were primarily financially motivated (Pande 2009). This is in contrast to the U.S., where surrogates (even those in commercial arrangements) cite altruism as their primary motivation (Ragoné 1994; Ciccarelli and Beckman 2005; Jadva et al. 2003). This worry of wrongful financial inducement is widespread. But, if a payment induces the desire to become a surrogate, it does not immediately follow that it is exploitation. If one makes a decision to do something merely because it will benefit her, which is the case for nearly all jobs that a person might accept, this doesn’t lead us to conclude that we should not pay her for that job or that we should pay her less (Crozier 2010; Humbyrd 2009; Wilkinson 2003). Coercion generally means that one threatens to make another worse off if they do not perform some act (Wertheimer 1992). Paying someone who voluntarily chooses to be a surrogate does not fall under this category. But, omissive coercion, Wilkinson adds, occurs when someone threatens to not benefit someone in a way that they are owed, unless they perform some act (2003). Since society, or the state, likely owes a woman some standard of welfare (survival at the minimum), and if surrogacy or something worse is the only way to achieve that standard, then she is essentially threatened with starvation if she doesn’t do it (Wilkinson 2003). Then, her consent would be invalidated by the fact that she is coerced into being a surrogate to get benefits which she is owed regardless, which would be exploitation.

However, as Wilkinson points out, any transaction could in principle be exploitative. The question is whether or not there is something inherent to surrogacy that makes it necessarily exploitative (Wilkinson 2003). This does not seem to be true, since even in the case of exploitative surrogacy given above, it is no more exploitative than other low-paying jobs like factory work (Crozier 2010; Humbyrd 2009; Wilkinson 2003). One could argue that surrogacy is different from other jobs because of the increased physical and psychological risk associated with surrogacy. But, this implies the assumption that women cannot weigh the risks of surrogacy against the benefits of payment (Humbyrd 2009), and it does not take into account the risks associated with poverty or with other jobs available to them (Purdy 1989). As one Indian surrogate explained in an interview (before the 2015 regulations were put in place), “This is not exploitation. Crushing glass for 15 h a day [earning $25 a month] is exploitation” (Haworth 2007).

If societal circumstances create a situation in which a person must resort to a job that they otherwise wouldn’t, then banning that option only makes the person worse off (Spar 2005; Wilkinson 2003). Instead of constraining poor women further, we should work to provide them with adequate social services and more options on the labor market so that their other alternatives might be more appealing than something dangerous or harmful (Andrews 1988; Crozier 2010). Lupton agrees, commenting, “[T]hose who are outraged by this approach should bear in mind that this is the natural consequence of an unequal society, and if we cannot save people from being poor it makes no sense to stop them from making sacrifices to alleviate their situation merely because we are appalled at the nature of those sacrifices” (1986, p. 151). It then becomes clear that the exploitation issue arises not because of the nature of commercial surrogacy, but because of the nature of an unequal society.

### The commodification argument against commercial surrogacy

Even if a surrogate is not exploited, because she freely consents to this option and is well-paid, it has been argued that commercial surrogacy is still wrong because it improperly treats reproductive labor, women, and children as commodities (Anderson 1990; Holder 1984; Radin 1987; Sandel 2013). We address these three forms of commodification separately. In general, if it would be unethical to apply market norms to the production, exchange, and use of a good, then it is not a commodity, and to treat it as such is to value it inappropriately, which degrades or corrupts it (Anderson 1990).

#### Commodification of women’s reproductive labor

Anderson argues that women’s reproductive labor is improperly treated as a commodity in commercial surrogacy, because the surrogate’s labor is alienated (1990). According to Anderson, the proper end of a pregnancy is an emotional bond between the mother and the baby, and so paying her to repress the formation of that relationship is wrong (1990). We would respond that it is clear that commercial surrogacy commodifies reproductive labor, but it is not clear that there is anything ethically problematic about this. Other kinds of labor are commodified, and this is not deemed as improper commodification. The argument that women’s reproductive labor is different in a relevant way rests on norms about what the proper ends of pregnancy and childbirth are—that the bond between a woman and the baby she births is somehow sacred or untouchable. But, those norms are either derived from some social convention (which could be countered with other social conventions) or from its essential nature (which rests on some metaphysical or religious view) neither of which hold (Sandel 2013). It is an example of an argument that rests on symbolic rhetoric rather than logical argumentation or evidence.

Clearly there is no way to be sure that reproductive labor is special in some way such that it is degraded if it is commodified. As Spar points out, this is merely an assertion, not a fact (2005). If moral limits to the market do exist, there should be good reasons for drawing the line in a particular place, and there are not good reasons for excluding reproductive labor from the market domain.

#### Commodification of women

It is well-established that human beings themselves are degraded if they are commodified. This rests on the Kantian argument that humans have an inherent dignity which must be respected, and in order to respect it, humans must be treated as ends in themselves, never as a means only. Anderson (and others) argue that commercial surrogacy commodifies—and therefore degrades—women themselves. One way this occurs is through the manipulation of the surrogate to the point of dehumanizing her (Anderson 1990). Pande has illustrated this trend in her reports from research interviews with surrogates in India. Before the 2015 ban, the surrogates were often restrained in hostels, where their eating, drinking, and exercise was overseen by the hostel leaders, and they were allowed to see their family once a week or less (Pande 2010). They were psychologically manipulated by being repeatedly told that they were disposable wombs, merely vessels for carrying the fetuses, that they should not form a bond with the child and would not be allowed to even look at the child after giving birth (Pande 2010). They had little say over what happened to them and their bodies throughout the surrogacy process, which reflected a gross disregard for their autonomy (Gupta 2012; Vincent and Aftandilian 2013). This manipulation and control of surrogates was done for the commercial benefit of the brokers, who made more money if they were able to produce a healthy child for the intended parents without any setbacks (Bailey 2011; Gupta 2012).

This kind of dehumanizing treatment of surrogates is degrading to women, since their interests and ends are not respected. It was the main reason for the Indian government to issue the 2015 letter. But, even though we agree self-evidently with the Indian authorities that degrading and manipulating women is unethical and should be stopped, we must ask the question whether this is a necessary consequence of commercial surrogacy? If commercial surrogacy can occur in HIC’s such as the U.S. without restraining the autonomy of surrogates and treating them as disposable resources, the same should be possible in India and around the globe. Surrogates were degraded by the rhetoric used by the hostel leaders and brokers, not the surrogacy process itself. The problem stems from the profit-seeking mechanisms governing the industry. While leaving the market totally subject to free-market norms may undoubtedly lead to a lack of respect for the interests of surrogates, regulations to ensure their interests are respected (rather than prohibition) could solve this.

#### Commodification of children

Anderson further argues that surrogacy improperly treats children as commodities. The surrogate creates the child with the intention to give it up, for monetary advantage, in the interests of herself rather than those of the child (Anderson 1990). Sandel agrees that there is something wrong with commercial surrogacy since it is analogous to baby-selling (2013). In the famous case of Baby M, in which the surrogate claimed that she had rights to the child after giving birth, the supreme court of New Jersey (U.S.A.) invalidated the contract on the grounds that it was “the sale of a child, or at the very least, the sale of a mother’s right to her child…There are, in a civilized society, some things that money cannot buy” (Matter of Baby M 1988, p. 1248).

This is another argument based on symbolic rhetoric rather than logical argumentation or evidence. Supporters of commercial surrogacy respond by resisting this analogy. They argue that the payment is only for the time, effort, pain, and risk that the surrogate undergoes in carrying and giving birth to the child (Lawrence 1991). This can be ensured by requiring that the surrogate is paid each month, regardless of the outcome of the pregnancy (as suggested by the GCRP in the Netherlands). Since the pregnancy is planned by both parties who have the child’s best interests at heart, then it is not the same as the sale of an existing unwanted child (Lupton 1986). Paying other people for services that enable one to create and deliver one’s (own) child is a normal part of procreation; one might also pay a doctor to deliver fertility hormones, artificially inseminate, or perform a needed C-section (Andrews 1988). Accordingly, there is no evidence to suggest that parents treat their children as products or commodities after paying for surrogacy (Tong 1990).

Even if children are not being bought and sold per se, part of the aversion to commercial surrogacy is the cultural conception that children are priceless, and that it is therefore distasteful to place a monetary value on them (Ragoné 1994). Altruism (thus, the gift rhetoric) seems like the only appropriate way to handle the exchange of something priceless (Shaw 2007). This, too, is an argument based in symbolic rhetoric rather than logical arguments or evidence. Payment and altruistic motivation are not mutually exclusive (Van Zyl and Walker 2013). So, the pricelessness of children can still be honored by altruistic intention even if the surrogate is paid.

### Benefits of commercial surrogacy

#### Avoiding exploitation by underpayment

Most opponents to commercial surrogacy still find altruistic surrogacy to be acceptable, or even praiseworthy (Annas 1988). While the image of a surrogate as a selfless, altruistic saint is heartwarming, it can lead one to be blinded to the ethical issues with altruistic surrogacy. Not paying surrogates for the risks and labor involved in surrogacy is arguably exploitative (Van Zyl and Walker 2015). The surrogate’s gift is so substantial, in the sense that it causes a lot of pain and discomfort, and also in the sense that it creates a human child. To not reciprocate in some way could potentially subject her to self-sacrifice (Van Zyl and Walker 2013). Self-sacrifice is morally unacceptable because it reinforces the idea that the needs of the intended parents are more important than those of the surrogate, which is exploitative (Badcock 1986).

Viewing surrogacy as a gift relationship can also “obscure, or at least shifts the attention away from, the fact that the [surrogate] incurs a number of obligations towards the intending parents and the [fetus]” (Van Zyl and Walker, 2013, p. 376). The intended parents may feel uncomfortable voicing their concerns since the surrogate is giving them such a substantial gift. On the other hand, if the surrogate takes her moral obligations seriously, then she is at the mercy of the intended parents (Van Zyl and Walker, 2013). This relationship, if solely based on trust, can be dangerous. Further, if the surrogate’s motivations are defined as purely altruistic, then it serves to reduce her bargaining power (Drabiak et al. 2007). Commercial surrogacy, when properly regulated, involves a contract which stipulates all the rights and responsibilities of each party, making it clear that the surrogate cannot harm the fetus, and that she deserves adequate compensation for her labor (Van Zyl and Walker 2015). This is not to say that altruistic surrogacy is inherently unethical, but, altruistic surrogacy as the required format is problematic. The option to draw up a contract and receive adequate payment should be made available to every surrogate, and if she voluntarily turns down payment, that is of course her privilege.

#### Autonomy of intended parents and surrogate

Commercial surrogacy is one way of creating the opportunity for intended parents to fulfill their wish for a child. This supports the concept of procreative choice or autonomy, a right protected by the Californian constitution, for example (Lawrence 1991). The fact that intended parents go to such lengths to engage in CBS and recruit surrogates in other countries, which can be risky, is a testament to the strength of their desire to procreate. Respecting autonomy would mean facilitating this desire in a safe way. It is apparent that, in many countries, restrictions on accessing assisted reproductive services are discriminatory towards people who are unmarried and/or LGBTQ + . The 2019 surrogacy regulation bill in India includes such restrictions, which demonstrates that the law is not only intended to protect surrogates but also to restrict procreative autonomy. Allowing commercial surrogacy for all desiring parents, and with it some kind of agency that recruits surrogates and pairs them with intended parents, would make it far easier for intended parents to achieve their procreative plans.

Allowing commercial surrogacy also promotes the autonomy of surrogates. It is sexist and paternalistic to assume that women cannot make the decision to engage in certain practices for money (Andrews 1988). It is therefore ironic that feminists have argued against commercial surrogacy on the basis of harm to women, when it has been central to the feminist movement “that women have a right to reproductive choice—to be able to contracept, abort, or get pregnant… to control their bodies during pregnancy... to create non-traditional family structures… These rights should not be overridden by possible symbolic harms or speculative risks” (Andrews 1988, p. 73).

The assertion that commercial surrogacy promotes autonomy for surrogates might seem contradictory to the claim that Indian surrogates are more likely to have their autonomy restricted by surrogacy brokers and economic duress. This is why we stress the importance of context in assessing the ethicality of commercial surrogacy. Bailey makes a good point that extending western moral frameworks, particularly those that focus on autonomy, choice, and liberalism, can erase or distort the experiences of subjects in non-western countries who may not place the same value on concepts like autonomy (2011). Pande points out that most Indian surrogates, up until 2015, in fact, downplayed the role of choice in their decision to become surrogates, by saying it is their motherly/familial duty (2010). While this may serve to minimize their role as money-makers for their family, it is one form of resistance that reinforces their self-worth (Pande 2010). Further, what might be viewed by outsiders as autonomy-restricting prisons, the surrogacy hostels were also seen as safe spaces where surrogates ccould gain skills for future employment, build networks with the women around them, and use their combined power to protect each other’s interests (Pande 2010). Many Indian surrogates also found the surrogacy process to be empowering, even if only because they could make enough money to lift themselves and their families out of poverty (Spar 2005). This is an important illustration of the complexity of the real-life experiences of surrogates, demonstrating that parts of their story may contain oppression and others empowerment. By engaging with the first-hand narrative of surrogates in India, prohibition of commercial surrogacy does not necessarily follow. Rather, proper regulations could have the potential to ensure that Indian surrogates are empowered rather than oppressed.

## Regulating payments for surrogacy in different contexts

### Surrogacy as labor

We have argued that it is not wrong to commodify women’s reproductive labor, and one of the reasons surrogates are exploited and wrongly commodified is because surrogacy is not treated as a legitimate form of labor. In India, where surrogacy used to be referred to as prostitution and stigmatized, the surrogates often had to hide the fact that they were becoming surrogates from their extended families and communities, and they reiterated their altruistic intentions and duties to avoid being considered selfish (Pande 2010). If reproductive work were seen as a legitimate avenue for earning money, the stigma and instrumentalization would be reduced. Surrogates might be viewed more like healthcare workers or temporary guardians than dehumanized incubators (Humbyrd 2009).

Van Zyl and Walker argue that the issues with altruistic and commercial surrogacy can be addressed by using the professional model (2013). In this model, it is accepted that surrogates might be motivated by their desire to offer a worthwhile service while still expecting to be paid. Professionals, such as teachers and nurses, share a strong ethical dimension to their work (Carr 1999), which requires them to harbor some internal motivation (beyond payment alone) to perform their job well. Surrogacy also contains this ethical dimension, which is one reason it is suitable to consider it a profession. Then protections can be granted by regulatory bodies that oversee surrogacy, similar to those overseeing other professions. But, professional unions would not be enough to govern surrogacy in the international market. Internationally upheld regulations to ensure surrogates are protected and well-paid in all places are necessary.

### Ethical payment in the Netherlands: minimum wage

In the Netherlands, the GCRP suggests that a fair maximum payment would be €500 per month, on top of immediate expenses, amounting to about €5000 total. This amount was calculated as a scaled-up version of what egg donors are paid for their time and effort in the Netherlands, which is €900 for one donation cycle. However, that number is not necessarily sufficient. In the U.S., surrogates are paid between US$10,000 and $40,000, while U.S. egg donors are paid around $4000 for one cycle (Covington and Gibbons, 2007). The egg donation payment guidelines were originally set as a scaled-up version of sperm donor compensation of $75–$100 per sperm sample (Krawiec 2014). Then, the compensation level for surrogates is arbitrary because it is far removed from the original deciding factor (the amount of time spent on a sperm donation).

The problem is that the discussion operates around a *maximum* payment in the first place. It has been established that coercion by high payment is possible in places with extreme financial inequality and lack of support for the very poor, but even in those situations, paying them less would actually be more exploitative. In the Netherlands, social welfare programs are adequate and background conditions are relatively fair. If we accept that surrogacy is a legitimate form of work, in line with the professional model proposed by (van Zyl and Walker 2013), then a *minimum* wage needs to be honored.

But, the GCRP still wants to keep surrogacy altruistic, while establishing the maximum payment per month as a suitable gift for reciprocating the altruism of the surrogate. It appears that the Dutch conception that surrogates should not be paid a livable wage stems from the concept – whether social, metaphysical, or religious—that reproductive labor is somehow special, and so it should not be commodified like other kinds of labor. But, as we have seen, this argument has no logical or evidential basis. Since underpayment is the only relevant ethical issue in the Netherlands, surrogates should be paid full-time minimum wage for every month that they are engaged in the surrogacy process—that includes the time before and after the pregnancy during which they undergo medical appointments, implantation, recovery, etc. Of course, this does not take into consideration the fact that the labor of surrogates occurs 24 h per day, not only during an 8-h workday. But, given that a surrogate can for the most part continue to do other activities during the pregnancy, it seems that full time (8-h per day) minimum wage would be sufficient to honor her efforts, since it would be the same amount she could make if she were to work a different job during this time. In the Netherlands, the minimum wage for persons over 22 years old is about €1600 per month (January 2019), which would result in a minimum payment of about €16,000 total for the pregnancy (equivalent to almost US$18,000).

In addition to paying the surrogates well, additional requirements for the protection of surrogates need to be in place to prevent ethical problems unrelated to payment. The GCRP suggests requirements such as independent legal representation for the surrogate, insurance policies (including life insurance) to be taken out in case of harm to the surrogate and/or to the intended parents, psychological/medical screening of the surrogate, and required counseling for the surrogate, before, during, and after the pregnancy (GCRP 2016, Ch. 11.4).

### Ethical payment in India: fair trade

Unethical payment by brokers and other third parties, who profited themselves as much as possible but exploited the surrogates paying them only a minimum amount of money, was a main reason for the Indian authorities to ban commercial surrogacy (cfr. Timms 2018). We agree with the authorities and other spokespeople that exploitation by third parties is unethical and should be stopped, but not by prohibiting commercial surrogacy outright. Ensuring ethical payment and treatment of surrogates in India, and other LMICs, is complicated but not impossible. CBS makes it unclear how much surrogates should be paid since the value of the payment is different for the intended parents than for the surrogate. While minimum wage might be an appropriate mechanism to ensure fair wages for surrogates in the Netherlands, it is not sufficient in India and many other LMICs. This is because minimum wage in India varies by region and industry, and some industries do not adhere to a minimum wage, such as the apparel and footwear industries (U.S. Department of State 2008). For example, the minimum wage for agriculture workers in Maharashtra, is only 100 INR (US$1.40) per day^2^ (GOI 2015), and 33% of India was making less than $1.25 per day in 2010 (Marriner 2012). This is remarkably low, considering that the international poverty line, under which a person is considered to be in extreme poverty, is US$1.90 per day (United Nations Sustainable Development Goals (SDGs). 2019). Finding solutions for widespread poverty and low wage-rates is beyond the scope of this paper, but exploitation should be avoided wherever possible. Particularly in the case of outsourced labor, workers in LMICs are exploited when they are paid much lower real wage rates^3^ than workers in HICs would be paid for the same work. These are the issues with the fertility industry that need to be addressed in regulating payments to surrogates.

One mechanism that is widely utilized to avoid exploitation of workers in the global labor market, particularly in the agriculture industry, is Fair Trade. According to The World Fair Trade Organization (World Fair Trade Organization (WFTO), 2017): "Fair Trade is a trading partnership, based on dialogue, transparency and respect, that seeks greater equity in international trade. It contributes to sustainable development by offering better trading conditions to, and securing the rights of, marginalized producers and workers—especially in the South.” Humbyrd suggests extending the mechanisms of Fair Trade to the international surrogacy market (2009).

The first principle of Fair Trade that is addressed by Humbyrd is payment of a fair price. This requires equivalent real wage rates to what surrogates are paid elsewhere, payments that are a justifiable proportion of what the fertility clinic/broker makes from the surrogacy, and payments given regardless of the outcome of the pregnancy. Fair payment, according to the WFTO, is at least the Local Living Wage. This minimum requirement to meet the principle of fair payment is in line with our suggestion of requiring minimum wage in HICs, which is (at least in principle) calculated in accordance with the cost of living in those HICs. Most surrogates in India were already paid more than a local living wage. Most of them made in 10 months as a surrogate more than they would have otherwise made in 3–15 years of work (Gupta 2012). The average amount of $5000 earned by a surrogate in India comes out to over 12 × the above-poverty-level wage of $1.90 per day.

However, Fair Trade is still necessary, since a living wage is not the only factor that makes it a fair wage. It also needs to be a “freely negotiated and mutually agreed wage,” and it needs to represent an “equitable share of the final price paid to each player in the supply chain” (WTFO). This means it is necessary that surrogates are a part of the discussion about how much they are going to be paid, which was not happening in India when commercial surrogacy was legal (Singh 2014). Regulations should require that the contract is translated and that direct interaction with the intended parents is permitted and facilitated. There should be a third-party organization, such as a professional union, which can process complaints from surrogates and can provide independent legal protection of surrogates, at the expense of the intended parents (Vincent and Aftandilian 2013). A fair wage also means that profits to clinics and brokers must not be raked in without sufficient benefit to the surrogates, and so some percentage of the total payment should be ensured to the surrogate. If a third-party protects the surrogates’ interests, payments to surrogates will occur regardless of the outcome of the pregnancy, and if her ability to negotiate her wage is ensured, then attempts to reduce the surrogate’s agency by instrumentalizing her will have no place.

Humbyrd suggests making Fair Trade a strict requirement for international surrogacy, which can be enforced through checks within the immigration system that must be utilized to bring home a child born to a surrogate abroad (2009). We agree with Humbyrd’s suggestion, but we think it should be extended such that the requirements are in place even within LMICs, not just for the case of CBS. This is why we have suggested, in line with Vincent and Aftandilian, a third-party organization which protects the surrogates’ interests within the country, and membership to this organization should be a requirement for becoming a surrogate (much like a professional union), and the costs would have to be paid by the intended parents. Because it is precisely the diversity of how different countries handle surrogacy remuneration that drives this practice abroad, Spar is right in suggesting an international agreement, which could extend principles from the Hague Convention on intercountry adoption (2005).

### Remaining issues ‘beyond the money’

As we have discussed, there is a form of exploitation that occurs when surrogates are coerced into becoming surrogates by their desperate financial situation. It has been established that this is not an issue inherent to surrogacy, but an issue with an unequal society (and by extension, global inequalities). One reality in India is that inequality is racially stratified, and people (especially women) with darker skin or those in “lower” castes are systematically disadvantaged (Singh 2014). The trend of outsourcing labor to poor countries occurs along race and class lines and thus perpetuates those distinctions on a global scale. International commercial surrogacy continues to be intertwined with unfair and racist background conditions globally, and this is not solved by regulating payments or surrogacy itself.

Bailey suggests using a reproductive justice approach in order to start the conversation about how to mitigate these ethical issues (2011). Reproductive justice does not necessarily have to come from the governments of individual countries. An international treaty governing commercial surrogacy can also require that part of the payment from intended parents goes towards capacity building and global projects in reducing inequality, maybe through the professional bodies that would oversee the regulation of surrogacy. This would hopefully open up more options for women so that the choice to become a surrogate can be freer. Allowing commercial surrogacy across nations would open up the possibility of finding a surrogate in any country, preferably one’s own country. Instead of banning commercial surrogacy outright and reserving surrogacy for Indian couples only, opening up commercial surrogacy globally might be a good alternative in order to avoid unethical exploitation. Then, given the better conditions for surrogates offered by other countries, surrogacy markets in LMIC’s might be pressured to reform, in a way that goes above and beyond the ability of outside regulation to reform it. So, while we do not find cross-border commercial surrogacy to be unethical in itself, we do think the remaining issues with the practice could be mitigated through the process of homogenizing the regulations across the world, which would in turn reduce CBS.

## Conclusion

Commercial surrogacy is not inherently unethical, but it can lead to certain issues that need to be addressed through regulations, and context is important in addressing those ethical issues. Exploitation by coercion is not an issue with commercial surrogacy but an issue with an unequal society/world, and it occurs in all forms of low-paying labor (particularly outsourced labor). Banning commercial surrogacy would not solve this, since it would only remove this opportunity for women to alleviate their poverty. The problem that needs to be addressed instead is the desperate nature of their decisions, which must be done through broader efforts to reduce inequality. Commodification of women’s reproductive labor is a non-issue. The claim that it is degrading to pay women for this kind of labor rests only on symbolic or religious norms and not logical or evidential ones. Commodification of children is a non-issue in surrogacy because payment for the reproductive labor is necessarily different from payments for existing children. The commodification of women is an issue that needs to be addressed, particularly where women are instrumentalized by the physical and mental manipulation that treats them as disposable resources for the benefit of the fertility industry. However, it is possible to respect the interests and ends of surrogates by treating them as laborers rather than non-human resources, given specific regulations.

Not only is commercial surrogacy justifiable when properly regulated, it can also be beneficial. It avoids the issue of exploitation by underpayment and it creates clarity in the obligations of both parties. It promotes reproductive autonomy of intended parents and empowers surrogates to choose what to do with their bodies and to profit from this choice. Commercial surrogacy should be properly regulated as a legitimate form of labor. We have suggested following the professional model for surrogacy. The surrogates’ interests and negotiating power should be protected by a local, independent professional body which they are required to join. Surrogates should be paid well, and payments should be given at regular intervals across the period of surrogacy and irrespective of the outcome. What counts as just payment depends on the context. In HICs such as the Netherlands, full time minimum wage is sufficient to ensure that surrogates are compensated for their work. In LMICs such as India, minimum-wage may not be sufficient, given that it is sometimes non-existent or below poverty-level, so the mechanism of Fair Trade should instead be used. This would mean that the surrogacy industry should be required to pay surrogates a fair living wage for their region, equivalent to the real-wage rate of what surrogates in the west are paid. The wage should be mutually agreed upon and freely negotiated, and the surrogate should get a fair portion of the payment paid to the agency/broker. This would avoid exploitation by underpayment. These requirements would also mitigate the wrongful commodification of women, since it would no longer be permitted, nor beneficial, to downplay their role as agents with interests. An international treaty that requires countries to regulate their surrogacy markets to protect surrogates, in line with minimum-wage or Fair Trade, is necessary. By opening up commercial surrogacy to the world, intended parents would be less likely to engage in CBS, and so self-regulation of the market will occur in combination with outside regulations. This legitimization of the surrogacy market and regulation to avoid exploitation and commodification of surrogates can go hand in hand with the reproductive justice approach, which would give women more agency in their lives so that their decision to become surrogates can be as free as possible.

## Footnotes

1 “Intended parents” refers to the party that recruits a surrogate to have a child for them, but this party may be a single person or a couple of any gender. This party is also known as the commissioning parents (particularly for commercial surrogacy), the desiring parents, or the wish-parents.

2 One of the reasons minimum wages are lower in particular industries is because of the inability of those industries to pay more, and regional differences are in part due to differences in cost of living (ILO 2018).

3 The real wage rate is adjusted for purchasing power, what can be bought in goods and services, with some amount of money, while the nominal wage rate is the actual amount in U.S. dollars.
